# Development and application of empowerment assessment tools for caregivers: a scoping review

**DOI:** 10.1002/alz.087300

**Published:** 2025-01-09

**Authors:** junhong wu

**Affiliations:** ^1^ Nanjing University of Chinese Medicine, nanjing, jiangsu China

## Abstract

**Background:**

To systematically analyze the current situation and application of caregiver empowerment assessment tools at home and abroad, to provide a reference for the future development of caregiver empowerment assessment tools and the assessment of caregiver empowerment level.

**Method:**

According to the research method of scope review, the relevant literature from the establishment of the database to October 2023 was searched, and the references of the included literature were traced, and the relevant literature of the caregiver empowerment assessment tool was comprehensively collected.

**Result:**

A total of 856 articles were retrieved. According to the inclusion and exclusion criteria, 14 articles were finally included. After data extraction, a total of 13 kinds of caregiver empowerment assessment tools were summarized, mainly from China and the United States. The application group was mainly the caregiver group with limited behavioral activities or cognitive impairment, and the overall reliability and validity level was good.

**Conclusion:**

There are many kinds of caregiver empowerment assessment tools at home and abroad. Different tools have their own scope of use. Nearly half of the tools have not been further applied, and the reliability and validity test indicators are different. Follow‐up researchers should pay attention to the full test of reliability and validity when developing the caregiver empowerment assessment tool, and evaluate it with recognized standards, focusing on the application and promotion of the tool.

